# Role of Intrinsic and Extrinsic Factors in the Regulation of the Mitotic Checkpoint Kinase Bub1

**DOI:** 10.1371/journal.pone.0144673

**Published:** 2015-12-10

**Authors:** Claudia Breit, Tanja Bange, Arsen Petrovic, John R. Weir, Franziska Müller, Doro Vogt, Andrea Musacchio

**Affiliations:** 1 Department of Mechanistic Cell Biology, Max Planck Institute of Molecular Physiology, Otto-Hahn-Straße 11, 44227, Dortmund, Germany; 2 Centre for Medical Biotechnology, Faculty of Biology, University Duisburg-Essen, Universitätsstrasse, 45141, Essen, Germany; Florida State University, UNITED STATES

## Abstract

The spindle assembly checkpoint (SAC) monitors microtubule attachment to kinetochores to ensure accurate sister chromatid segregation during mitosis. The SAC members Bub1 and BubR1 are paralogs that underwent significant functional specializations during evolution. We report an in-depth characterization of the kinase domains of Bub1 and BubR1. BubR1 kinase domain binds nucleotides but is unable to deliver catalytic activity *in vitro*. Conversely, Bub1 is an active kinase regulated by intra-molecular phosphorylation at the P+1 loop. The crystal structure of the phosphorylated Bub1 kinase domain illustrates a hitherto unknown conformation of the P+1 loop docked into the active site of the Bub1 kinase. Both Bub1 and BubR1 bind Bub3 constitutively. A hydrodynamic characterization of Bub1:Bub3 and BubR1:Bub3 demonstrates both complexes to have 1:1 stoichiometry, with no additional oligomerization. Conversely, Bub1:Bub3 and BubR1:Bub3 combine to form a heterotetramer. Neither BubR1:Bub3 nor Knl1, the kinetochore receptor of Bub1:Bub3, modulate the kinase activity of Bub1 *in vitro*, suggesting autonomous regulation of the Bub1 kinase domain. We complement our study with an analysis of the Bub1 substrates. Our results contribute to the mechanistic characterization of a crucial cell cycle checkpoint.

## Introduction

The spindle assembly checkpoint (SAC) is a safety mechanism that monitors the attachment of kinetochores to spindle microtubules and stalls mitotic progression by inhibiting the anaphase promoting complex/cyclosome (APC/C) until chromosome bi-orientation along the mitotic spindle is achieved [1–3]. SAC signaling takes place, at least in part, at kinetochores, large proteinaceous structures that mediate the interaction of chromosomes with the microtubules of the mitotic spindle [1].

**Fig 1:**
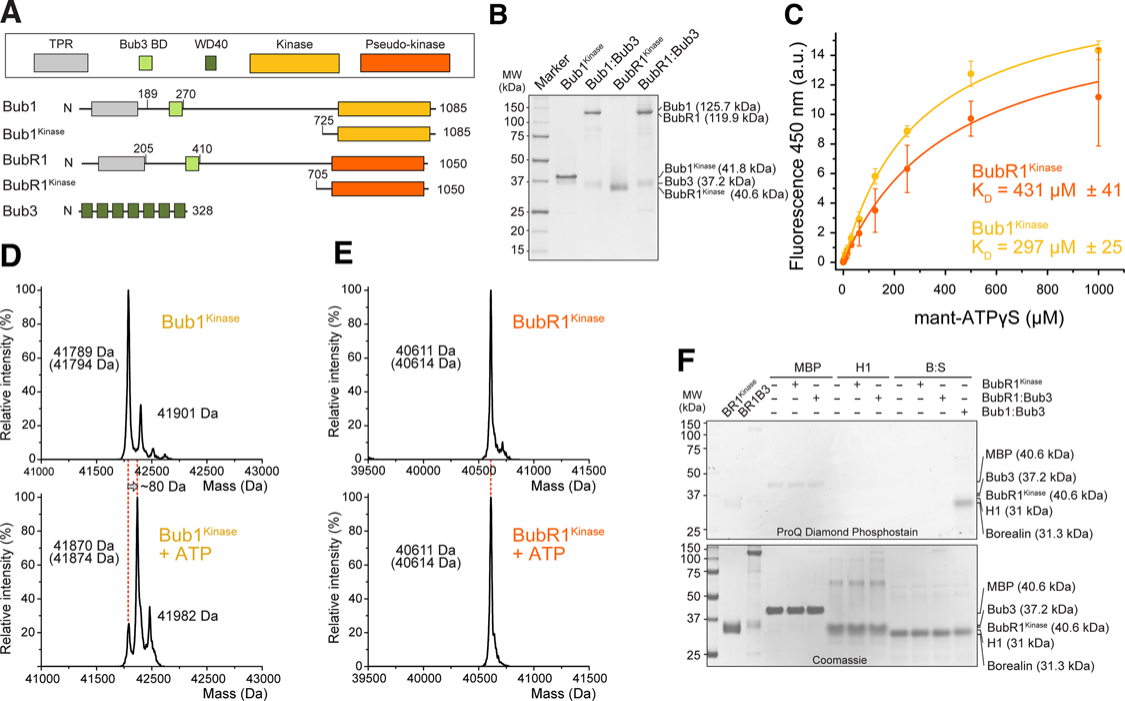
Reconstitution of Bub1 kinase and BubR1 pseudo-kinase. (**A**) Bub1 and BubR1 share a similar domain organization. Schematic description of the domains and constructs used in this manuscript: TPR–tetratricopeptide repeat, BD–binding domain, WD40 –an approximately 40-residue sequence repeat often terminating with a tryptophan (W) and aspartate (D) dipeptide. (**B**) Purified Bub1^kinase^, Bub1:Bub3, BubR1^kinase^, and BubR1:Bub3 were separated by SDS-PAGE. Their respective expected molecular mass is indicated. (**C**) Both Bub1^kinase^ and BubR1^kinase^ bind to mant-ATP. The change in fluorescence emission at 450 nm is plotted as a function of total mant-ATP concentration. The data were fitted with a one site binding equation using Origin 9.0, with R^2^ = 0.99 for both curves. Error bars represent SD of a mean of at least 2 independent experiments. a.u.–arbitrary units. (**D**-**E**) ESI-MS spectra of purified Bub1^kinase^ (**D**) or BubR1^kinase^ (**E**) before and after incubation with ATP for auto-phosphorylation. Theoretical calculated masses are given in brackets under the measured masses. (**F**) BubR1 is not an active kinase. Maltose binding protein (MBP), H1, and the Borealin:Survivin complex (Bor:Sur) were incubated with 50 nM BubR1 constructs and ATP and analyzed on SDS PAGE visualizing phosphates using the Pro-Q® Diamond Phosphoprotein Gel Stain. 10 nM Bub1:Bub3 was used as a positive control. BR1^kinase^–BubR1^kinase^, BR1B3 –BubR1:Bub3.

Bub1 and BubR1 are paralogous proteins at the core of the SAC. They probably evolved from a singleton (i.e. a progenitor gene) already present in the last eukaryotic common ancestor (LECA). Several independent duplication events occurred during speciation, in all cases followed by substantial sub-functionalization [4, 5]. In mammals, Bub1 and BubR1 share similar domain organizations (Fig 1A) [6, 7]. Their function in the SAC, however, is clearly distinct. BubR1 is a crucial component of the checkpoint effector, the mitotic checkpoint complex (MCC) that directly inhibits the APC/C [8–10]. Bub1, in contrast, is not part of the MCC. After becoming recruited to kinetochores in prometaphase, Bub1 contributes to recruiting additional SAC proteins, including Mad1, Mad2, BubR1, Bub3 and Cdc20, thereby promoting the incorporation of a subset of them into the MCC [11–17].

It has been proposed that BubR1 kinase activity is required for SAC function and for an additional role of BubR1 in chromosome alignment [18–21]. Other studies, however, demonstrated BubR1 kinase activity to be dispensable for the spindle checkpoint and chromosome alignment [22–26]. More recently, it was proposed that BubR1 is an inactive pseudo-kinase whose kinase domain (BubR1^kinase^) contributes to the overall stability of the protein [4]. This may explain previous discrepancies, in particular because it was shown that point mutations normally utilized to disrupt kinase activity largely decreased the overall structural stability of BubR1 [4].

Contrarily to BubR1, Bub1 is a *bona fide* kinase [27–29], but its activity is believed to play a marginal role in the SAC [12, 30–34]. In addition to its role in the SAC, Bub1 is also required for chromosome congression and alignment [14, 35]. This function of Bub1 requires its kinase activity [30, 34, 36, 37]. Bub1 phosphorylates Thr120 of histone H2A (P-T120-H2A), which in turn promotes centromere accumulation of Shugoshin and protein phosphatase 2A (PP2A), thus contributing to the establishment and protection of centromeric cohesion as well as to Aurora B kinase recruitment [30, 37–45].

How Bub1 kinase is regulated on a molecular level remains unclear. Intra-molecular regulation by the N-terminal TPR domain (Fig 1A) was shown to contribute to kinase activation [36, 46], but another study did not confirm this [29]. Structure determination of the Bub1 kinase domain (Bub1^Kinase^) showed that the P+1 loop, a short motif that follows the activation loop and that contributes to substrate recognition, creates a steric obstruction expected to prevent effective access of substrates to the active site [27] (PDB ID 4R8Q). The P+1 loop, however, undergoes a profound rearrangement following phosphorylation, with the latter ultimately relieving the auto-inhibited conformation and activating Bub1 [29] (PDB ID 4QPM). Conversely, there is no evidence that phosphorylation of the activation loop, which is crucial for the activation of many kinases [47, 48], plays a role in the case of Bub1.

In this study, we set out to assess the mechanism of Bub1 regulation and thoroughly characterized known interactions of Bub1 at the kinetochore and their implications for Bub1 kinase activity *in vitro*. In order to resolve conflicting reports of kinase activity, we compared Bub1 and BubR1 activities and established that BubR1 is not an active kinase. After reconstitution and biochemical characterization of Bub1:Bub3 complexes with BubR1:Bub3 and Knl1, we conclude that these binding partners do not alter Bub1 kinase activity. Furthermore, we find that Bub1 substrates share a weak common sequence motif and that DNA binding might additionally aid in directing Bub1 to nucleosomes. Finally, we confirm the phosphorylation of the P+1 loop on S969 (P-S969) and demonstrate that this auto-phosphorylation is crucial in conferring Bub1 activity.

## Results

### BubR1 is an inactive pseudokinase

We expressed recombinant versions of the BubR1 and Bub1 kinase domains (indicated as BubR1^kinase^ and Bub1^kinase^, respectively) as well as the full-length versions of Bub1:Bub3 and BubR1:Bub3 complexes in insect cells and purified them to homogeneity (Fig 1B). All four recombinant samples eluted as a single peak from size-exclusion chromatography (SEC) columns (which separate proteins based on size and shape), suggesting they are monodisperse (S1A Fig. The profile for Bub1:Bub3 and BubR1:Bub3 are shown in Fig 5A). “Classical” kinase inactivating mutations such as K821R and K795R in Bub1 and BubR1, respectively, resulted in dramatically reduced yields of expressed and purified product (data not shown), in line with the idea that these mutations have pervasive effects on kinase stability that transcend their potential deleterious effects on enzymatic activity [4]. Conversely, mutation of the catalytic aspartate (D) of Bub1 (D917) to asparagine (D917N mutant) did not apparently perturb the stability of Bub1 (not shown).

To test if these domains interact with nucleotides, we titrated Bub1^kinase^ and BubR1^kinase^ with increasing amounts of 2'-/3'-O-(N'-Methylanthraniloyl)-ATP (mant-ATP) [49], a nucleotide-analog whose fluorescence emission at 450 nm changes upon modifications of the fluorophore’s environment associated with incorporation in the kinase active site. BubR1^kinase^ and Bub1^kinase^ bound to mant-ATP with similar dissociation constants, strongly suggesting that BubR1^kinase^ has not lost its ability to bind ATP, contrary to recent predictions [4, 50] (Fig 1C). The relatively low affinity of BubR1^kinase^ and Bub1^kinase^ for mant-ATP in comparison to previous measurements with other kinases [e.g. reference [51]] probably reflects an unfavorable interaction of the mant group with the Bub1 and BubR1 scaffold that reduces the overall binding affinity for the modified nucleotide. Nevertheless, the similarity of binding affinity for the Bub1 and BubR1 domains suggests that they are both competent to bind nucleotides. Furthermore, we were able to release mant-ATP from the kinase active site with an ATP analog (S1B and S1C Fig)

Incubation of Bub1^kinase^ with ATP, followed by whole-protein mass spectrometry analysis (ESI-MS), revealed an auto-phosphorylation event that led to an increase in the mass of the protein corresponding roughly to the addition of a single phosphate group (Fig 1D). Conversely, this was not observed when BubR1^kinase^ was incubated in the presence of ATP (Fig 1E). Furthermore, we failed to detect phosphorylation of established kinase substrates such as MBP, histone H1, and the Borealin:Survivin complex (Bor:Sur) by BubR1^kinase^ or the BubR1:Bub3 complex, while Bub1:Bub3 successfully phosphorylated Bor:Sur even when using a 5-fold lower kinase to substrate ratio in the phosphorylation reaction. (Fig 1F). In agreement with a recent proposal [4], these data show that Bub1^kinase^ and Bub1:Bub3 are active kinases, while BubR1^kinase^ and BubR1:Bub3 are not, even despite the ability of BubR1^kinase^ to bind ATP.

### Bub1^kinase^ is an active enzyme that undergoes auto-phosphorylation

**Fig 2:**
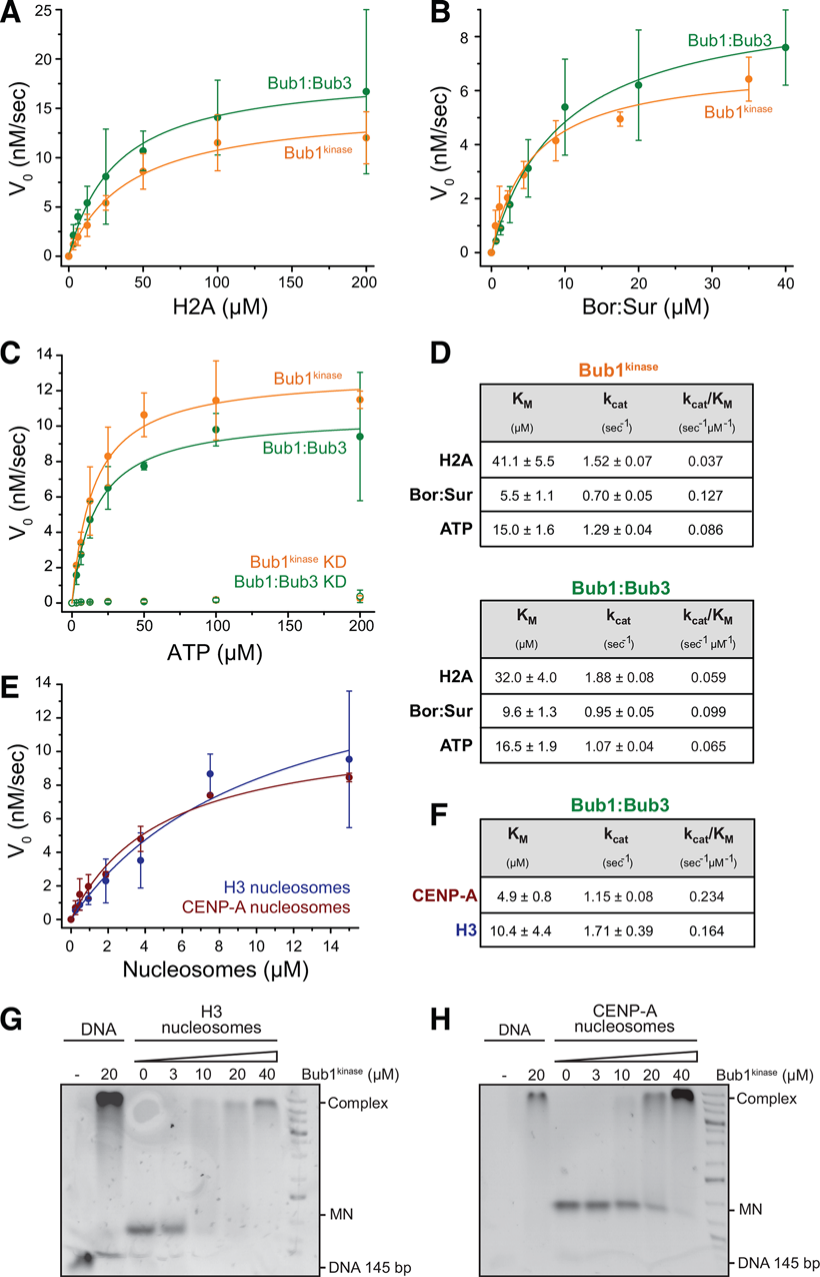
Kinetic characterization of Bub1 complexes. (**A**-**C**) Bub1^kinase^ and Bub1:Bub3 complex exhibit similar catalytic activity toward H2A (**A**) and Bor:Sur substrates (**B**) and hydrolyze ATP at similar rates (**C**). The mutation D917N abrogates ATPase activity (**C**). The kinase activity was determined using the ADP-Glo^TM^ Kinase Assay and is plotted as a function of substrate concentration to allow fitting according to the Michaelis-Menten equation with R^2^ = 0.99 (Bub1^kinase^ on Bor:Sur R^2^ = 0.97). KD–kinase dead. Error bars represent SD of a mean of at least 2 independent experiments. (**D**) Kinetic parameters of the Michaelis-Menten fits as determined in (**A**-**C**). (**E**) H2A contained in H3- or CENP-A nucleosomes is efficiently phosphorylated by Bub1^kinase^ and Bub1:Bub3. (**F**) The kinase activity, plotted as a function of substrate concentration, allows fitting with the Michaelis-Menten equation with R^2^ = 0.95 (H3) and 0.99 (CENP-A). Error bars represent SD of a mean of at least two independent experiments. (**G-H**) EMSA assays showing DNA and nucleosome binding of Bub1^kinase^ and H3- or CENP-A nucleosomes. MN–mononucleosomes.

The ADP-Glo^TM^ Kinase Assay (Promega) measures a luminescent signal that is correlated linearly with ADP generated in an ATP phosphotransferase reaction, thus permitting time-dependent monitoring of kinase reactions at different concentrations of protein substrates and of ATP. Bub1^kinase^ and Bub1:Bub3 were tested in this assay against recombinant histone H2A or the Bor:Sur complex. Initial reaction velocities were measured at several substrate concentrations and at a fixed concentration of ATP (200 μM) (Fig 2A and 2B). In separate experiments, ATP concentration was varied at a fixed concentration of substrate (200 μM) (Fig 2C). Bub1^kinase^ and Bub1:Bub3 phosphorylated H2A and Borealin efficiently at largely substoichiometric concentrations of kinase (10 nM). Data fitting with the Michaelis-Menten equation identified the catalytic parameters (K_M_ and k_cat_), collected in Fig 2D.

The catalytic efficiency (k_cat_/K_M_) of Bub1^kinase^ and Bub1:Bub3 emerging from this analysis (in the order of 0.1 sec^-1^μM^-1^) are very similar, arguing that Bub1 may not be regulated by regions outside the kinase domain or by Bub3. We note that both in terms of K_M_, and of catalytic efficiency (k_cat_/K_M_), H2A is a worse Bub1 substrate than Borealin in the Borealin:Survivin complex.

### Bub1^kinase^ binding to nucleosomes enhances H2A phosphorylation

We were surprised to see that at least *in vitro* the Bor:Sur complex was a better substrate than H2A, and wondered if phosphorylation of nucleosomes, rather than isolated H2A, was more efficient. We therefore compared the ability of Bub1:Bub3 or Bub1^kinase^ to phosphorylate free H2A or H2A incorporated in nucleosomes that also contained either histone H3 or its centromeric variant CENP-A. Measurements of the initial velocity of phosphorylation at different substrate concentrations revealed ~3 to 6 fold lower K_M_s for H2A in nucleosomes in comparison to free H2A, with an up to 4-fold overall enhancement of catalytic efficiency (Fig 2E and 2F). Even in the presence of nucleosomes, the reaction appeared specific for H2A, as shown in experiments at saturation (S2A Fig), although we cannot exclude phosphorylation of histone H2B, whose size is almost identical to that of H2A. Our analysis also shows that Bub1 phosphorylates H2A in nucleosomes reconstituted with CENP-A at least as well as it phosphorylates H2A in nucleosomes reconstituted with canonical histone H3. Given that P-T120-H2A is enriched at kinetochores [e.g. see reference [29]] it is not implausible that this modification might occur on CENP-A-containing nucleosomes.

Preference for nucleosomes as substrates for Bub1 activity might suggest that H2A in isolation lacks features implicated in recognition by Bub1 and that the phosphorylation of H2A could be enhanced by nucleosome binding of Bub1. We therefore tested if Bub1^kinase^ binds nucleosomes in an electrophoretic mobility shift assay (EMSA). We used both the auto-phosphorylated and the dephosphorylated forms of Bub1^kinase^ and either CENP-A- or H3-containing nucleosomes or free DNA. After mixing Bub1^kinase^ with mononucleosomes (at a concentration of 0.5 μM), we observed a Bub1-concentration-dependent mobility shift, indicative of complex formation. Bub1^kinase^ bound CENP-A or H3-nucleosomes with similar apparent affinity (Fig 2G and 2H); furthermore, binding was independent of the phosphorylation status of Bub1^kinase^ (S2B Fig). Bub1^kinase^ also readily bound to free DNA, which might provide an explanation for improved binding of Bub1 to nucleosomes as compared to free H2A (Fig 2G and 2H).

### P-S969 is crucial for kinase activity

Bub1 may regulate its activity via auto-phosphorylation [29]. When incubated with 1 mM ATP for 16 hours, Bub1^kinase^ auto-phosphorylates (Fig 1D). LC-MS/MS spectra demonstrated S969 to be the only prominently phosphorylated residue in Bub1^kinase^ (not shown), in agreement with a recent study [29].

Previous studies revealed the structures of the unphosphorylated [27] and the auto-phosphorylated [29] forms of Bub1^kinase^, both bound to ADP and 2 Mg^2+^ ions coordinated to the α and β phosphates of ATP [PDB ID codes 4R8Q and 4QPM, respectively. Note that 4R8Q is the result of a re-refinement of the structure previously deposited with the PDB code 3E7E, in which ATP, which had been originally modeled in the structure [27], was replaced with ADP and a second Mg^2+^ ion [29]].

**Fig 3:**
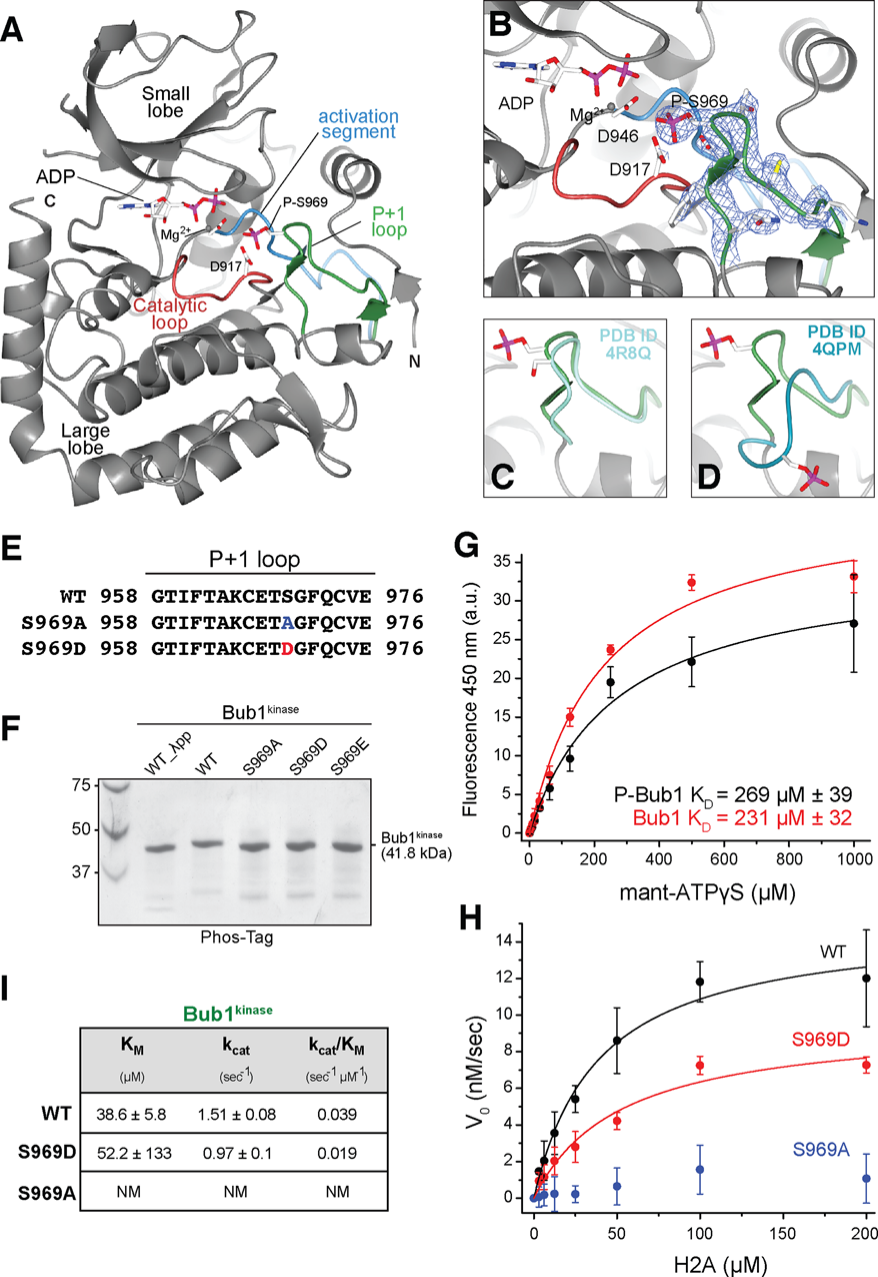
S969 is a major phosphorylation site in Bub1^kinase^. (**A**) Ribbon diagram showing the model of phosphorylated Bub1^kinase^. N and C indicate the N- and C-terminus, respectively. The P+1 loop harboring the phosphorylated S969 is highlighted in green, the activation loop is in blue, and the catalytic loop in red. Side chains of residues D946, D917, S969 and ADP are shown as sticks, an Mg^2+^ atom is represented by a gray sphere. (**B**) Detailed view of the active site of Bub1 showing P-S969 pointing towards the catalytic aspartate at position 917 (D917) and ADP. The electron density around the P+1 loop represents a 2F_o_-F_c_ map contoured at 1.5σ as a blue mesh. (**C**-**D**) Overlay of phosphorylated Bub1 with unphosphorylated Bub1 [4R8Q, (**C**)] and phosphorylated Bub1 [4QPM, (**D**)]. The P+1 loop of 4R8Q and 4QPM is colored in shades of blue. All images were created with CCP4MG [53]. (**E**) Amino acid sequence of the Bub1 P+1 loop with the mutations of S969 highlighted in red (S969D) and blue (S969A). (**F**) Bub1^kinase^ wild-type (WT) and S969A, S969D, S969E were incubated with ATP or λ-phosphatase (λ-pp) and analyzed on SDS PAGE using Phos-tag to detect a phosphorylation-specific shift. (**G**) P-S969 does not interfere with ATP binding of Bub1^kinase^. The change in fluorescence emission at 450 nm is plotted as a function of total mant-ATPγS concentration. The data were fitted with a one site binding equation using Origin 9.0 and R^2^ = 0.99. Error bars represent SD of a mean of at least two independent experiments. a.u.-arbitrary units. (**H**) The phosphomimetic S969D is able to restore kinase activity while S969A is catalytically inactive. The kinase activity is plotted as a function of substrate concentration to allow fitting according to Michaelis-Menten kinetics with R^2^ = 0.99 (WT), R^2^ = 0.97 (S969D). Error bars represent SD of a mean of at least 2 independent experiments.

We obtained diffraction-quality crystals of P-S969-Bub1^kinase^ in a new crystal form (Table 1) and determined the structure by molecular replacement at a resolution of 2.4 Å using the atomic model of unphosphorylated Bub1 (PDB ID 3E7E) [27] as a search model (Table 1). Expectedly, the final model of P-S969-Bub1^kinase^ is closely related to that of the previously reported unphosphorylated Bub1^kinase^ and P-S969-Bub1^kinase^ (overall r.m.s of 0.75 Å for 327 Cα positions (PDB 4R8Q) and 0.68 Å for 324 Cα positions (PDB 4QPM) [27, 29] (Fig 3A). Like other members of the eukaryotic protein kinase family, Bub1 consists of a small N-terminal lobe (N-lobe) rich in β-sheet structure, and a larger C-terminal lobe (C-lobe) rich in α-helices. The ATP binding cleft is located at the interface of the two lobes. The activation segment, which comprises the magnesium-binding loop, the activation loop, and the P+1 loop, is a prominent feature at the entry site of the catalytic cleft [48]. It creates a platform for substrate recruitment and its presentation to the catalytic apparatus and to ATP (Fig 3A).

**Table 1:** Data Collection and Refinement Statistics.

***Data Collection***	
Wavelength (Å)	1.0000 Å
Resolution range (Å)	43.3–2.4 (2.486–2.4)
Space group	P 1 21 1
Unit cell (a, b, c, α, β, γ)	62.38 66.34 106.54 90 98.34 90
Total reflections	233252 (23340)
Unique reflections	33866 (3332)
Multiplicity	6.9 (7.0)
Completeness (%)	99.85 (99.70)
Mean I/σ(I)	11.24 (1.03)
Wilson B-factor	56.36
R-merge	0.1133 (2.202)
R-meas	0.1227
CC1/2	0.999 (0.592)
CC*	1 (0.863)
***Refinement Statistics***	
Reflections used for R-free	1694
R-work	0.211 (0.386)
R-free	0.245 (0.406)
Number of non-hydrogen atoms	5606
- Macromolecules	5526
- Ligands	56
- Water	24
- Protein residues	680
Missing residues	A/B: 725–734; 807–815; 1084–1085
RMS (bonds)	0.004
RMS (angles)	0.853
Ramachandran favored (%)*	98
Ramachandran outliers (%)*	0
Clashscore*	4.49
Average B-factor	78.6
Macromolecules	78.9
Ligands	63.10
Solvent	50.60

Data for the highest-resolution shell are given in parentheses.

*R*_*merge*_ = Σ_*h*_Σ_*i*_|*I*_*h*,*i*_ − ⟨*I*_*h*_⟩|/Σ_*h*_Σ_*i*_ *I*_*h*,*i*_, where the outer sum (h) is over the unique reflections and the inner sum (i) is over the set of independent observations of each unique reflection.

*R*_*work*_ = ∑_*hkl*_||*F*_*obs*_| − |*F*_*calc*_||/∑*F*_*obs*_, where F_obs_ and F_calc_ are the observed and calculated structure factors of the respective reflections hkl.

R_free_ is equivalent to R_work_ but is calculated on a random set of reflections corresponding to 5% of all reflections and that are excluded from refinement.

*As defined by MolProbity [52]

**Fig 4:**
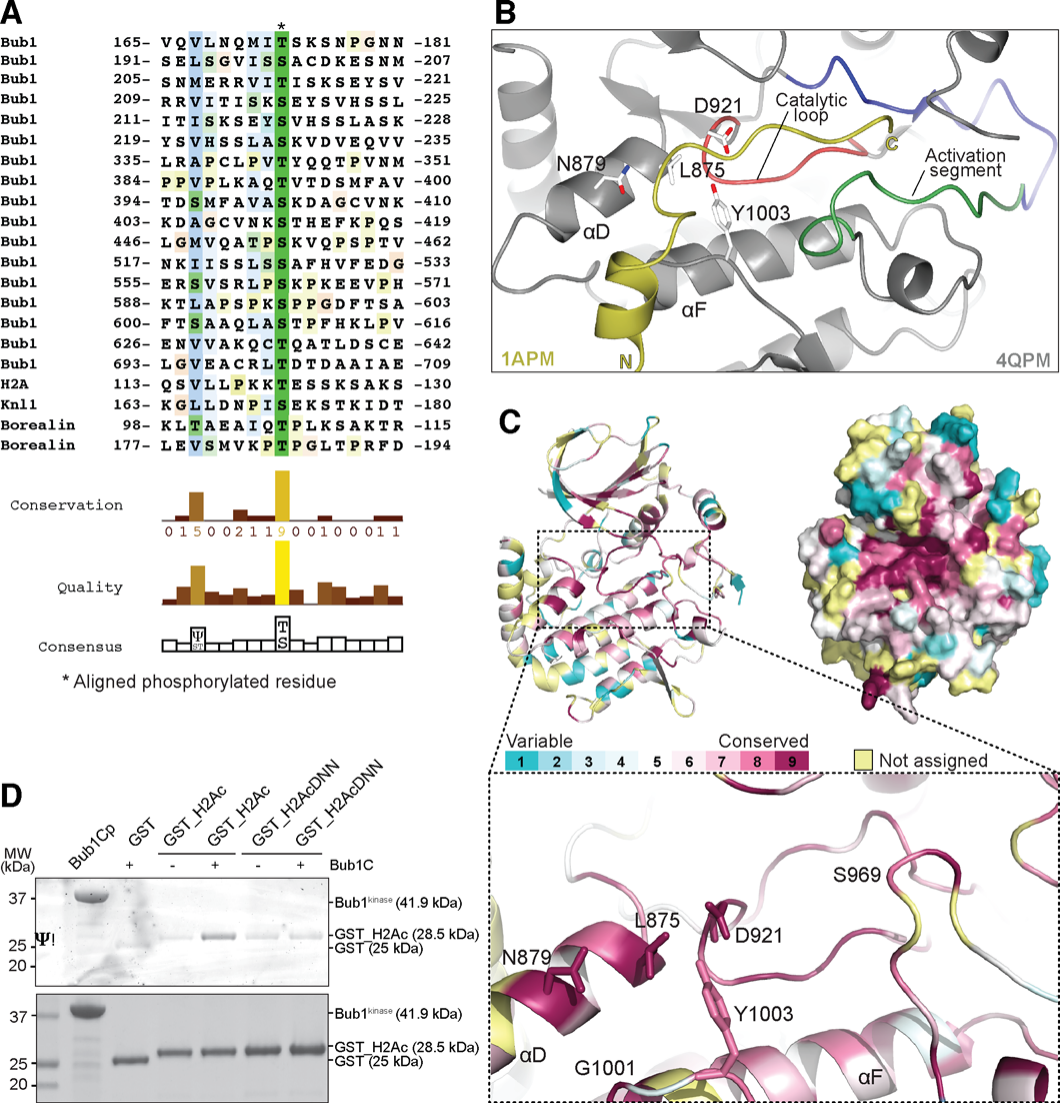
Bub1^kinase^ possesses conserved residues that recognize a substrate consensus sequence. (**A**) Alignment of phosphorylation sites found in Bub1-dependent phosphorylation reactions using the ClustalX algorithm in Jalview [54]. Numbers indicate boundaries of the protein segments shown; phosphorylated residues are denoted with an asterisk, the conservation of residues is highlighted using the ClustalX coloring scheme. (**B**) The structure of Bub1^kinase^ (PDB ID 4QPM) was superimposed onto the structure of PKA bound to a pseudo-substrate inhibitor [PDB ID 1APM, reference [55]]. The image shows only Bub1 and the position of the pseudo-substrate peptide (yellow) after alignment of PKA to Bub1. Bub1 residues putatively involved in substrate recognition are depicted as sticks. (**C**) Bub1^kinase^ sequence and structure conservation. The same residues as in (**B**) are shown in sticks. Conservation was determined by aligning Bub1^kinase^ from 14 organisms with ConSurf [56], the scoring legend is depicted on the upper left. Conservation scores obtained for positions in the alignment that had less than 6 un-gapped amino acids were considered to be unreliable and colored light yellow in the graphic visualization output. Images were created with CCP4MG [53] and Pymol (Schrödinger LLC, Portland, OR). (**D**) Bub1^kinase^-dependent H2A phosphorylation can be reduced by mutating V115D, L116N, L117N on H2A. GST-H2A constructs were incubated with Bub1^kinase^ and ATP, then analyzed by SDS PAGE, and phosphorylated proteins were specifically stained using Pro-Q® Diamond Phosphoprotein Gel Stain.

The conformation of the P+1 loop revealed by our structure, for which there is very clear density throughout (Fig 3B and S3A–S3C Fig), is clearly distinct from that previously reported for P-969-Bub1^kinase^ [29] and more similar to that observed for the unphosphorylated P+1 loop (Fig 3C and 3D). The phosphate group of pS969 points directly towards the co-crystallized ADP molecule, positioning the P+1 loop in such a way that it would be expected to prevent the ingression of a substrate peptide (Fig 3B, see also Fig 4B). The position of the phosphate on P-S969 is very close to that expected for the γ-phosphate should ATP, instead of ADP, sit in the binding site. In this position, the phosphate faces a highly negatively charged environment created by the directly neighboring residues E967 and D917 (Fig 3B). Electrostatic repulsion would be expected to extrude the phosphorylated P+1 loop, possibly explaining the conformation demonstrated by the recently published structure of P-969-Bub1^kinase^ [29] (Fig 3D). Thus, we suspect that the conformation we have trapped is stabilized by contacts with symmetry mates in the crystal.

We verified the positions of the P+1 loops of the phosphorylated and unphosphorylated Bub1 structures by refining our model of the P-969-Bub1^kinase^, after molecular replacement, against the deposited structure factors of the 4R8Q and 4QPM datasets (S3A–S3C Fig). After model rebuilding, the conformation of the P+1 loop essentially converged to the conformations of the 4R8Q and 4QPM reported previously [27, 29]. Collectively, the three structures likely depict Bub1 at different stages of the auto-phosphorylation process, and are consistent with the idea that phosphorylation at P-S969 is required to remove a steric blockade of the active site by the P+1 loop.

We then asked if phosphorylation of S969 was a prerequisite for kinase activity. To this end, we created alanine, glutamate, or aspartate mutants of S969 in Bub1^kinase^ (Fig 3E) After incubation with ATP and subsequent SDS-PAGE analysis, no phosphorylation-dependent mobility shift could be detected for the mutant or dephosphorylated Bub1^kinase^, in contrast to the wild-type protein (Fig 3F). This agrees with the idea that S969 is the major auto-phosphorylation site in the kinase domain. To test whether phosphorylation of S969 affects nucleotide binding by Bub1^kinase^, we compared ATP binding of phosphorylated and dephosphorylated Bub1^kinase^. Titrating mant-ATPγS against phosphorylated and dephosphorylated Bub1^kinase^ did not show significant change in the dissociation constants (K_D_) of ATP (Fig 3G), demonstrating that phosphorylation of S969 does not grossly affect ATP binding.

As S969 is located in the P+1 loop, which is involved in substrate binding, we expected to see a difference in the efficiency of substrate binding by the mutant kinases. We therefore assessed the activity of the different mutants on the H2A substrate (Fig 3H). While the phosphomimetic S969D and S969E were able to phosphorylate H2A efficiently, the H2A phosphorylation activity of the S969A mutant was almost entirely ablated (Fig 3H, S4A Fig). In conclusion, our results show that auto-phosphorylation at S969 is required for efficient peptide targeting during catalysis.

### Bub1 kinase possesses conserved residues that recognize a substrate consensus sequence

As the target specificity of Bub1 kinase remains largely unknown, we set out to test several kinetochore proteins as substrates for Bub1 kinase *in vitro*. Potential substrates were initially identified in phosphorylation reactions after SDS PAGE analysis and phosphorylation-directed staining with the Pro-Q Diamond phosphostain reagent. Substrates were further evaluated by LC-MS/MS to determine the sites of phosphorylation.

Along with the established substrate H2A, we identified Borealin (see above), Knl1, and Bub1 itself as targets of Bub1 activity (Fig 4A and data not shown). Alignment of the specific phosphorylation sites of different substrates allows the identification of the putative consensus motif ψ(x)_5_T/S, where ψ is an aliphatic residue and x is any amino acid (Fig 4A). We note that many of the substrates contain additional small aliphatic residues upstream of the acceptor site that may also contribute to specific recognition by Bub1 kinase, as well as potential additional phosphorylation acceptor sites at positions +2 or +3 relative to the acceptor.

Our attempts to crystallize Bub1^kinase^ with substrate peptides did not yield diffracting crystals to date. We therefore superimposed the coordinates of “open” Bub1 (4QPM) to those of cAMP-dependent protein kinase (PKA) bound to a pseudo-substrate inhibitor complex (PDB ID 1APM, Fig 4B), thus modeling the approximate position of a putative substrate peptide onto Bub1. We further aligned Bub1 sequences from 12 organisms from yeast to human using ConSurf [56] to elucidate the extent of sequence and structure conservation of Bub1 kinase (Fig 4C). This analysis pointed to strongly evolutionarily conserved residues such as L875 and N879 (in the αD helix), D921 (in the catalytic loop), G1001 and Y1003 (in the αF helix) of Bub1^kinase^ for a possible role in the stabilization and specificity of substrate recognition. (Fig 4C) [57]. To test this prediction, we introduced several point mutations in the Bub1^kinase^ domain, thus creating the L875A-N879A and G1001A-Y1003A double point mutants, and the L875A-N879A-G1001A-Y1003A quadruple point mutant. Solubility and stability during purification of the mutants were indistinguishable from those of wild type Bub1^kinase^, suggesting that the mutations do not grossly perturb the kinase domain (data not shown). This was clearly confirmed by the ability of the mutants to auto-phosphorylate to levels comparable to those of wild type Bub1^kinase^ (S5A Fig). We therefore tested the activity of these mutants on a GST-H2A substrate, which behaves essentially identically to full-length H2A as a Bub1 substrate (S4B Fig). In line with our prediction, all three mutants were unable to phosphorylate GST-H2A, even at very high substrate concentrations (S5B and S5C Fig). From our model of the complex of the PKA pseudo-substrate inhibitor with Bub1^kinase^, we surmise that these conserved residues of Bub1, whose mutation prevents substrate recognition, may interact with substrate residues at positions -6 to -4 relative to the acceptor site. To test this idea, we introduced the non-conservative mutations V115D, L116N, L117N in H2A and analyzed the potential of this ‘DNN’ mutant as a Bub1 substrate *in vitro*. In agreement with our idea, the DNN-mutant was a distinctly worse substrate in comparison to wild-type H2A (Fig 4D).

### Characterization of Bub1 complexes

**Fig 5:**
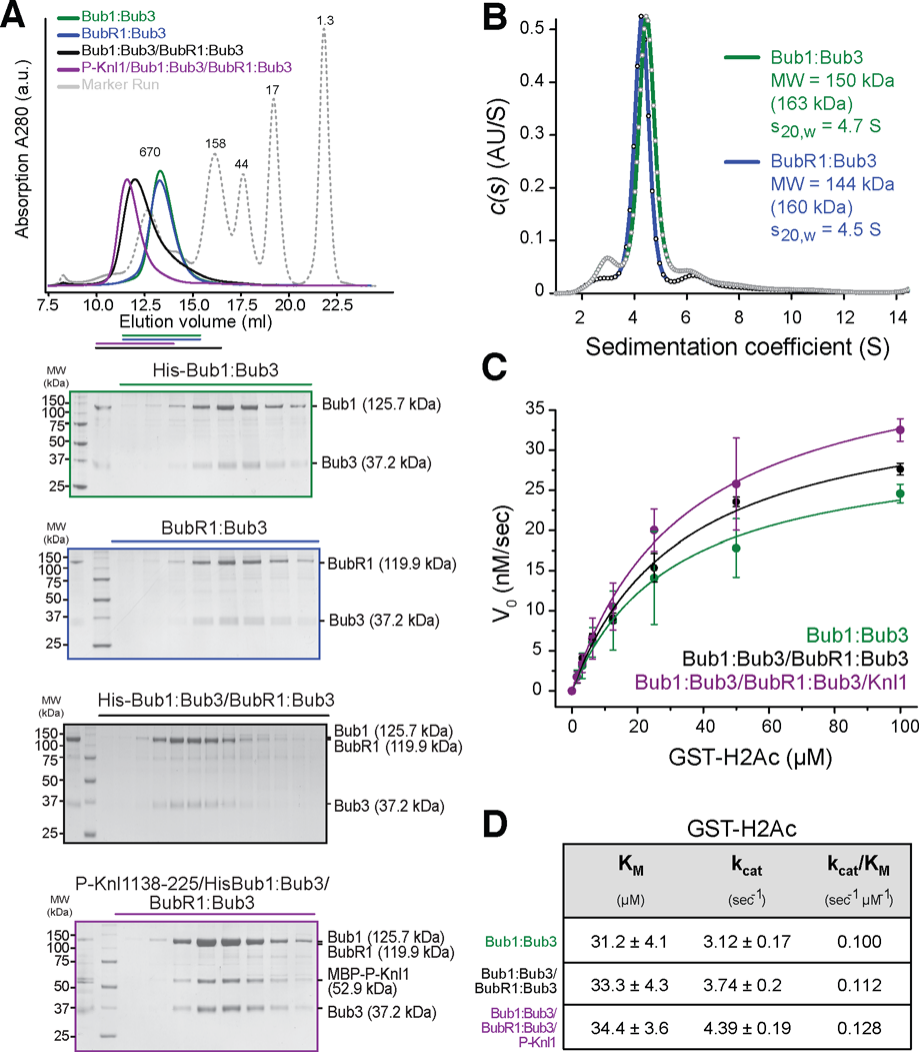
Biochemical characterization of Bub1 complexes. (**A**) Bub1:Bub3 (green) and BubR1:Bub3 (blue) co-elute in a stable complex from a size exclusion chromatography column (black) and can be assembled as a complex onto phosphorylated MBP-Knl1^138-225^ (purple). A bar of the respective color indicates fractions analyzed on SDS PAGE (below). (**B**) Analytical ultracentrifugation. Normalized c(s) distribution curves for Bub1:Bub3 (green) and BubR1:Bub3 (blue). A predominant peak at 4.5 S (s_20,w_ = 4.7) is apparent for Bub1:Bub3 and at 4.3 S (s_20,w_ = 4.5) for BubR1:Bub3 indicating a single dominant sedimentation species that corresponds to a theoretical molecular weight of 150 kDa and 144 kDa, respectively. The theoretical mass of a 1:1 complex is indicated for both in brackets. Frictional ratios were determined as 2.16 for Bub1:Bub3 and 2.21 for BubR1:Bub3. (**C**) Complexes of Bub1 exhibit similar kinase activity toward a GST-H2A substrate. The kinase activity is plotted as a function of substrate concentration to allow fitting according to the Michaelis-Menten equation with R^2^ = 0.99. Error bars represent SD of a mean of at least two independent experiments. (**D**) Kinetic parameters of the Michaelis-Menten fits as determined in (**C**). a.u.-arbitrary units.

Having established the role of auto-phosphorylation of the Bub1 kinase domain as an intra-molecular or intrinsic means of regulation, we investigated whether extrinsic factors can also modulate kinase activity. Recombinant full-length Bub1:Bub3 and BubR1:Bub3 complexes (Fig 1B) eluted nearly identically from a size exclusion chromatography (SEC) Superose 6 column (Fig 5A). The complexes eluted earlier than expected based on their theoretical mass, suggesting non-globular shapes, additional oligomerization, or both. To determine the effective state of oligomerization of the complexes, we resorted to sedimentation velocity analytical ultracentrifugation (AUC) experiments. The Bub1:Bub3 and BubR1:Bub3 complexes showed nearly identical behavior. The calculated molecular masses for Bub1:Bub3 (150 kDa) and BubR1:Bub3 (144 kDa) are in excellent agreement with those predicted for 1:1 complexes of each subunit (Fig 5B and S6 Fig).

It has been proposed that Bub1 and BubR1 interact directly and that this promotes the recruitment of BubR1 to kinetochore-bound Bub1 [15, 58, 59]. This interaction has not been reconstituted with full-length proteins, and therefore we asked if Bub1:Bub3 and BubR1:Bub3 formed a stable complex in a SEC experiment. The shift in the elution peak and co-migration of Bub1 and BubR1 bands on SDS-PAGE clearly demonstrated that the two full-length complexes interact tightly in a supra-molecular assembly (Fig 5A).

The interaction of the Bub1:Bub3 complex with the kinetochore subunit Knl1 is mediated by phosphorylation of the latter by the SAC kinase Mps1 [60–63]. This interaction is promoted by the direct binding of the Bub3 seven-WD40 β-propeller structure to phosphorylated Met-Glu-Leu-Thr (MELT) repeats of Knl1, and Bub1 contributes decisively to this interaction via a short motif named the “loop”. The equivalent “loop” region of BubR1 is unable to direct Bub3 towards phosphorylated MELT repeats (P-MELT), but, as explained in the previous paragraph, BubR1:Bub3 may be recruited via dimerization with Bub1:Bub3 [15, 61].

We asked if we could recapitulate these interactions with purified components. Indeed, Bub1:Bub3-BubR1:Bub3 complex bound a construct corresponding to residues 138–225 of Knl1 (Knl1^138-225^) that had been pre-phosphorylated with Mps1 kinase. Binding to phosphorylated Knl1 produced an additional shift and a remarkable compaction of the peak containing Bub1:Bub3 and BubR1:Bub3. Furthermore, SDS-PAGE analysis identified Knl1 as a stoichiometric component of the peak fractions (Fig 5A). Collectively, these results lend strong support to a model in which Bub1:Bub3 can interact concomitantly with P-Knl1 and BubR1:Bub3 at the kinetochore.

The availability of reconstituted forms allowed us to test whether BubR1:Bub3 or P-Knl1 binding modulate the kinase activity of Bub1:Bub3. Kinase activity against a GST-H2A tail-peptide fusion was tested with the ADP-Glo^TM^ Kinase Assay (Promega). In both cases, we did not detect major changes of Bub1 kinase activity upon complex formation (Fig 5D). Collectively, these observations are consistent with previous findings [29] and argue that Bub1 kinase activity, at least *in vitro*, is not regulated by extrinsic factors.

## Discussion

Bub1 and BubR1 reflect sub-functionalizations of paralogs generated by gene duplication [4, 5]. It is of extreme interest that several independent duplication and sub-functionalization events might have taken place in the course of evolution [4, 5]. To what extent the biochemical evolution of the components of the paralogous pairs that were created by these distinct duplications followed similar or different paths is currently unclear. The investigation of this very interesting question may contribute to the understanding of how the SAC network constrains the evolution of its components. This study moved from our desire to shed light on this question by investigating in biochemical detail the regulation of human Bub1 and BubR1. Our analysis of the BubR1 kinase domain is consistent with a recent report demonstrating that it is an inactive pseudo-kinase [4]. We extend this previous analysis by showing that the BubR1 kinase domain binds to nucleotides with an affinity comparable to that of Bub1. We note that this finding contradicts a recent study predicting that BubR1 might be unable to bind ATP [50, 64]. Thus, our results suggest that significant sequence divergence from active kinases in the Gly-rich loop (a region of kinases implicated in the stabilization of nucleotide binding) of BubR1, previously indicated as a possible cause of loss of activity [4], does not affect the ability of BubR1 to bind nucleotides. This suggests that divergence of BubR1 from Bub1 at other regions of the kinase domain, possibly in the catalytic loop [4], may be the predominant cause of its impaired activity.

Bub1, on the other hand, is an active kinase that hydrolyzes ATP for phospho-transfer to a variety of substrates with catalytic efficiencies, reported here, that are in line with those of many other kinases. One of the motivations behind this study was to explore whether the interactions of Bub1 with its most direct binding partners in the kinetochore, Bub3, Knl1, and BubR1, imposes an extrinsic level of regulation on Bub1 kinase activity. We conclude from our analysis that this may not be the case, at least when reconstituting interactions with recombinant material as described here. Previously, we, and others had reported that the TPR region might contribute to the regulation of the kinase activity of Bub1 [36, 46]. While our new analysis seems inconsistent with this earlier claim, it remains possible that the discrepancy may be generated by intrinsic differences in the Bub1 material utilized in these assays, as in our previous study we had used a precipitated form of Bub1 from HeLa cells as the source of kinase activity, whereas here we have used only recombinant material. A recent study also failed to identify differences in the kinase activity of full-length Bub1 and a TPR-deleted construct [29]. Yet another study, which was published while this work was under review, also reached the conclusion that Bub1 is not controlled by extrinsic factors and proposed that Bub1 auto-phosphorylation at Thr589, upstream of the kinase domain, is crucial for its activity [65]. Future studies will have to address the relative weight of Bub1 auto-phosphorylation at different target sites of its primary structure on the Bub1 activation mechanism.

If our analysis of candidate “extrinsic” regulators of the kinase activity of Bub1 did not unravel significant modulations, it represents nevertheless a very significant advancement in the reconstitution of interactions of SAC proteins. Our hydrodynamic analysis demonstrates for the first time that Bub1:Bub3 and BubR1:Bub3 form monodisperse 1:1 complexes devoid of further oligomerization. These complexes then interact in a tight ~300 kDa Bub1:Bub3/BubR1:Bub3 heterotetramer, which undergoes significant compaction when further combined with a segment of Knl1 containing a single (Mps1-generated) P-MELT repeat and two previously identified KI motifs, which interact with the TPR regions of Bub1 and BubR1 [46, 66–68]. These important results are in line with a model we have recently proposed for the role of Bub1:Bub3 as a receptor for kinetochore recruitment of BubR1:Bub3 [15]. Collectively, our results strongly suggest that the kinase activity associated with BubR1 precipitates in previous studies was Bub1’s [4, 20].

Phosphorylation of the activation segment leading to a structural rearrangement and hence activation is a common feature of protein kinases [48]. In Bub1, phosphorylation takes place on the P+1 loop, whose name reflects an involvement in the recognition of substrate downstream from the acceptor site. In agreement with a recent study [29], we identify an auto-phosphorylation site on the substrate-binding P+1 loop of Bub1, an atypical position for activating auto-phosphorylation in kinases. Our crystal structure of the phosphorylated kinase captured the P+1 loop in a conformation that creates a steric blockade to the active site, with the phosphate attached to the side chain of S969 pointing towards the nucleotide-binding pocket of Bub1. Our structure adds to two previous structures of the catalytic domain of Bub1 with the unphosphorylated and the phosphorylated P+1 loop [27, 29]. It provides what appears to be an intermediate conformation of the P+1 loop after phosphate transfer, before extrusion from the active site. A previous study reported that the choice of N-terminal boundary influences the catalytic activity of the Bub1 kinase domain [29]. A shorter construct (Bub1^740-1085^, whose structure is described by the 4QPM PDB dataset) was significantly more active and prone to auto-phosphorylating than a longer one (Bub1^724-1085^, whose structure is described by the 4R8Q PDB dataset). The boundaries of the construct used here (Bub1^725-1085^) are very similar to those of the less active (longer) construct. Structural comparisons show that the longer constructs form an additional short β-strand (engaging residues 735–738, which are absent in the shorter construct), whose presence might be surmised to stabilize a closed conformation of the P+1 loop, thus influencing kinase activity. While we have not carried out equivalent activity comparisons, the relatively long incubation time of 16 hours in our auto-phosphorylation reaction was clearly sufficient for complete auto-phosphorylation.[29].

We show that S969 is most likely the only phosphorylation site on the Bub1 kinase domain and that it is critical for Bub1 activity. The conservation of this residue highlights the necessity of a negatively charged residue at this position for Bub1 activity. In *S*. *cerevisiae*, the serine is replaced by an aspartate at the equivalent position, circumventing a requirement for phosphorylation. The level of phosphorylation of S969 does not appear to change during the cell cycle [29], but the overall levels of Bub1 drop rapidly after mitosis [58, 69].

Collectively, the structural and biochemical analysis of Bub1 indicate that it is a constitutively active kinase, as further suggested by the presence of an intact R-spine, a structural motif within the kinase domain and a hallmark of active kinases, in Bub1 [29, 70]. This has implications for the local and temporal regulation of Bub1 activity. Even being inherently active, Bub1 may need to be specifically recruited to kinetochores (by Knl1) in prometaphase to phosphorylate its physiologically relevant substrates at the kinetochore and centromere. As a result of restricted localization, during mitosis Bub1 is positioned close to its substrates and its presence there is further restricted in time, implying spatial and temporal control of its kinase activity. Indeed, H2A is only found phosphorylated in mitosis [37, 40].

Numerous kinetochore substrates have been reported for Bub1 kinase, amongst which Mad1, Bub3, Incenp, Cdc20, H2A, and Bub1 itself [28, 29, 37, 42, 71, 72]. We show here that also Knl1 and Borealin are Bub1 substrates *in vitro*. The phosphorylation sites on Knl1 are interesting because they precede the KI1 motif of Knl1, which binds the TPR domain of Bub1 [46, 66–68]. Borealin, which is a subunit of the chromosome passenger complex and whose function is to anchor Aurora B at centromeres, is strongly phosphorylated in mitosis [73–75], but the role of these phosphorylation sites and their relevance *in vivo* remains to be established. Our analysis of substrates reveals a possible consensus motif for Bub1, and mutations in H2A at some of the positions we have identified abolish phosphorylation by Bub1. Overlay of kinase-peptide structures with the Bub1 structure identified a highly conserved surface on Bub1 whose position is compatible with a role in substrate recognition, a hypothesis that we confirmed experimentally. A recent paper identified residues in the αC helix and in the P+1 loop (defined as αC1, αC3 and APE -7 residues) as additional determinants of specificity within the kinase family [57]. Here, we did not focus on these determinants of specificity because they likely contribute to recognition of substrate residues downstream from the acceptor site, whereas the most conserved features of the consensus substrate we discuss occur upstream of the acceptor site. However, we note that phosphorylation of S969, which neighbors the APE-7 position, has clearly the potential to influence substrate recognition.

Taken together, these contributions significantly extend our understanding of the mechanism of specific substrate recognition by Bub1 kinase.

## Methods

### Expression and purification of His_6_-Bub1:Bub3 and His_6_-BubR1:Bub3 and BubR1^kinase^ (BubR1 residues 705–1050) constructs

His_6_-Bub1:Bub3, His_6_-BubR1:Bub3 and BubR1^kinase^ constructs were obtained by cloning the coding sequences for Bub1, BubR1 and Bub3 into pFLMultiBac vector and generating infectious viruses [76]. Expression was carried out by infection of Tnao38 insect cells [77] or Sf9 cells (Invitrogen) at 27°C for 72 hr. Insect cells were harvested by centrifugation at 5900xg for 20 min in a Sorvall RC 3BP+ (Thermo Scientific) centrifuge with Rotor H6000A. The pellets were re-suspended in 10 volumes of lysis buffer (50 mM Tris pH 8, 300 mM KCl, 2 mM MgCl_2_, and 2 mM PMSF). Cells were lysed by sonication and cleared by centrifugation at 108800xg, 4°C (Rotor JA 30.50, Avanti J-30I, Beckman Coulter). The lysate was filtered using Rotilabo® syringe filters 0.45 μm before being passed on a 5 ml HisTrap FF (GE Healthcare). Fractions containing protein were pooled diluted to 50 mM KCl and either directly purified by anion exchange chromatography (Resource Q 6 ml, GE Healthcare), or first incubated with TEV protease overnight or for 4 h. The fractions containing the protein were further purified by size exclusion chromatography on a S200 10/300 or S75 16/60 column (GE Healthcare) in 50 mM Tris pH 8, 150 mM KCl, 2 mM DTE, 2 mM MgCl_2_. Peak fractions were pooled, concentrated to typically 5–10 mg/ml, frozen in small aliquots in liquid nitrogen, and stored at −80°C.

### Expression and purification of Bub1^kinase^ (Bub1 residues 725–1085) constructs

Mutations of GST-Bub1^kinase^ constructs were obtained by site-directed mutagenesis using the coding sequence for Bub1 cloned into pFLMultiBac vector and generating infectious viruses [76]. Expression was carried out by infection of Tnao38 insect cells [77] at 27°C for 72 hr. Insect cells were harvested by centrifugation at 5900xg for 20 min in a Sorvall RC 3BP+ (Thermo Scientific) centrifuge with Rotor H6000A. The pellets were re-suspended in 10 volumes of lysis buffer (50 mM Tris pH 7.6, 50 mM NaCl, 2 mM MgCl_2_, 1mM DTE and 2 mM PMSF). Cells were lysed by sonication and cleared by centrifugation at 108800 x g, 4°C (Rotor JA 30.50, Avanti J-30I, Beckman Coulter). The lysate was filtered using Rotilabo® syringe filters 0.45 μm before being passed on a 1 ml glutathione resin (Amintra Expedeon Inc). The protein was eluted with 20 mM glutathione, fractions containing protein were proteolyzed with PreScission protease overnight to eliminate the GST tag, and then subjected to auto-phosphorylation by adding 1 mM ATP, or to dephosphorylation by adding λ-phosphatase. After tag cleavage, the Bub1 protein was passed on anion exchange chromatography (Resource Q 6 ml, GE Healthcare), the flow-through was concentrated and further purified by size exclusion chromatography on a S75 16/60 column (GE Healthcare) in 25 mM Tris pH 7.6, 100 mM KCl, 2 mM TCEP, 10 mM MgCl_2_. Peak fractions were pooled, concentrated to typically 10–15 mg/ml, frozen in small aliquots in liquid nitrogen, and stored at −80°C.

### Nucleotide binding

Binding of nucleotides was assessed by measuring the change in fluorescence emission of a mant-labeled nucleotide upon kinase binding. Mant-labeled nucleotide was titrated from 0–1 mM to the kinase using 1 μM kinase in all measurements. Fluorescence emission at 450 nm (excitation at 340 nm) was recorded using a microplate reader Infinity M200 (Tecan); the emission of mant-nucleotide alone was subtracted from the emission of the complex to obtain a binding isotherm. Data were fitted to a one site binding model equation using the Origin 9.0 software (OriginLab Corp., Northampton, USA) to determine binding parameters.

### Crystallization and structure determination

Initial conditions were obtained using 5 mg/ml protein and 2 mM ADP in a buffer containing 25 mM Hepes 7.6, 100 mM NaCl, 2 mM TCEP, 10 mM MgCl_2_, with a Mosquito® Nanodispenser (TTP LabTech Ltd.) by the sitting-drop method. The conditions were refined to obtain diffraction-quality diamond-shaped crystals of 200 μm diameter after two days at 4°C. The reservoir solution contained 15% PEG 3350 and 0.2 M NaCl, crystals were cryoprotected in this reservoir solution, supplemented with 20% (v/v) glycerol and then flash-frozen in liquid nitrogen. Diffraction data were collected from crystals cooled to 100 K at the beamline X10SA of the Swiss Light Source (Paul Scherrer Institute, Villigen, Switzerland) at a wavelength of 1 Å. Images were recorded on a Pilatus 6M detector, indexed and integrated using XDS and scaled using XSCALE [78]. Initial phases of the phosphorylated kinase were obtained by molecular replacement using PHASER [79] with the coordinates of human Bub1 kinase (PDB ID 3E7E, now superseded by 4R8Q) [27] as a search model. The structure of the Bub1 kinase was then further refined using rigid-body refinement and iterative rounds of restrained refinement using PHENIX [80], interspersed with manual rebuilding using Coot [81]. The final model was validated using MolProbity [52] Graphical representation was generated using CCP4MG [53] or Pymol (Schrödinger LLC, Portland, OR). The final model contains two Bub1 monomers (including residues 735–806 and 816–1083 for chain A and B), two ADP molecules and two magnesium atoms.

### Kinase Assay

The ADP-Glo^TM^ Kinase Assay was implemented to allow for the quantitative measurement of ADP produced in the kinase reaction. The kinase reaction was carried out by mixing a first substrate, concentrations from 0 to 200 μM, with a second substrate at saturating concentration 200 μM in kinase buffer (25 mM Tris pH 7.6, 10 mM MgCl_2_, 150 mM NaCl, 5 mM TCEP) and starting the reaction by the addition of kinase. Luminescence was recorded using the microplate reader Infinity M200 (Tecan). The luminescence produced in the assay correlated linearly with the amount of ADP generated by the kinase. Taking different time points of the reaction allowed the determination of initial velocities of the kinase reaction that were plotted as a function of the substrate concentrations used. The obtained data plots were fitted to the Michaelis-Menten equation and analyzed using the Origin 9.0 software (OriginLab Corp., Northampton, USA) to determine catalytic parameters.

### Sedimentation velocity analytical ultracentrifugation

Sedimentation velocity experiments were performed in an Optima XL-A analytical ultracentrifuge (Beckman Coulter, Palo Alto, US-CA) with Epon charcoal-filled double-sector quartz cells and an An-60 Ti rotor (Beckman Coulter, Palo Alto, US-CA). Samples were dialyzed against buffer (50 mM Tris pH 8, 150 mM KCl, 3 mM MgCl2, 2 mM TCEP) that was used as a reference. Samples were centrifuged at 203,000xg at 20°C and 500 radial absorbance scans at 280 nm were collected with a time interval of 1 min. Data were analyzed using the SEDFIT software [82] in terms of continuous distribution function of sedimentation coefficients (c(S)). The protein partial specific volume was estimated from the amino acid sequence using the program SEDNTERP. Data were plotted using the program GUSSI comprised in the SEDFIT software [82].

### EMSA

Nucleosome binding of Bub1 was analyzed by EMSA. 0.5 μM nucleosomes were incubated for 30 min with 0–40 μM Bub1 on ice in 22.5 mM Hepes pH 7.5, 90 mM NaCl, 1.8 mM TCEP, 4.5 mM MgCl_2_, 1 mM AppNHp. The complexes were separated using a 0.7% agarose gel, run in 0.2% TBE, at 20 mM for about 2 h using free DNA and unbound protein as references.

### Phosphorylation-site analysis

Liquid chromatography coupled with mass spectrometry was used to identify phosphorylation sites. Proteins of interest (~10 μg) were incubated with 10 nM kinase in buffer (25 mM Tris pH 7.6, 10 mM MgCl_2_, 150 mM NaCl, 5 mM TCEP) and 0.5 mM ATP for at least 4 h at 25°C. Samples were then digested with LysC/Trypsin and/or GluC and prepared for LC-MS/MS analysis as previously described [83]. 100 ng of peptides were separated on a Thermo Scientific^TM^ EASY-nLC 1000 HPLC system (Thermo Fisher Scientific^TM^, Odense, Denmark); in an one hour gradient from 5–60% acetonitrile with 0.1% formic acid and directly sprayed via a nano-electrospray source in a quadrupole Orbitrap mass spectrometer (Q Exactive^TM^, Thermo Fisher Scientific^TM^) [84]. The Q Exactive^TM^ was operated in data-dependent mode acquiring one survey scan and subsequently ten MS/MS scans [85]. Resulting raw files were processed with the MaxQuant software (version 1.5.2.18) using a reduced database containing only the proteins of interest for the search and giving phosphorylation on serine, threonine and tyrosine as variable modification. [86]. A false discovery rate cut off of 1% was applied at the peptide and protein levels and the phosphorylation site decoy fraction.

## Supporting Information

S1 Fig SEC elution profiles of Bub1 and BubR1. **(A**) Bub1^kinase^ (yellow) and BubR1^kinase^ (orange) elute from a Superdex 200 size exclusion chromatography column in a single peak and as expected for globular proteins. A molecular weight standard is shown in gray. a.u.–arbitrary units. (**B**) Binding of Bub1^kinase^ and BubR1^kinase^ to mant-ATP (as in Fig 1C). (**C**) After achieving maximal binding of mant-ATP (1 mM), an ATP analog, AMP-PCP, was added at the indicated concentrations, resulting in dose-dependent reduction of the mant-ATP fluorescence signal.
(EPS)

S2 Fig Nucleosome binding of phosphorylated Bub1^kinase^. (**A**) Bub1^kinase^ and Bub1:Bub3 full-length complex efficiently phosphorylate H2A in H3- or CENP-A nucleosomes or as free H2A. (**B**) EMSA showing DNA and nucleosome binding of phosphorylated Bub1^kinase^ and H3- or CENP-A nucleosomes. MN–mononucleosomes.
(EPS)

S3 Fig Bub1 structures have a clearly defined density around the P+1 loop. Detailed view of the P+1 loop of (**A**) phosphorylated Bub1^740-1085^ (PDB ID 4QPM), (**B**) unphosphorylated Bub1^724-1085^ (PDB ID 4R8Q), and (**C**) Bub1^kinase^ (5DMZ, this study). Side chains are shown as sticks. The electron density around the P+1 loop for 4QPM and 4R8Q has been rebuilt and refined from the structure factors and is represented by the 2F_o_-F_c_ map, contoured at 1.5σ as a blue mesh for all structures.
(TIF)

S4 Fig Bub1^kinase^ S969E is an active enzyme while S969A is less efficient. (**A**) H2A phosphorylation was analyzed on SDS PAGE after incubation with Bub1^kinase^, Bub1^kinase^ S969E, or Bub1^kinase^ S969A, and ATP using Pro-Q® Diamond Phosphoprotein Gel Stain. Auto-phosphorylation of Bub1^kinase^ was used as a positive control. (**B**) GST_H2Ac (H2A 113–130) is a substrate for Bub1^kinase^ while GST is not. H2A and auto-phosphorylation of Bub1 were used as positive controls.
(EPS)

S5 Fig Kinetic analysis of Bub1 mutants predicted to affect substrate recognition. (**A**) The L875A-N879A (LN), G1001A-Y1003A (GY), and L875A-N879A-G1001A-Y1003A (LNGY) mutants of Bub1^kinase^ underwent auto-phosphorylation to levels comparable to those of wild type Bub1^kinase^, as indicated by the Pro-Q® Diamond Phosphoprotein Gel Stain. Note that the LN, GY, and LNGY mutants were used in these experiments as fusions to GST. (**B**) The activity of the L875A-N879A (LN), G1001A-Y1003A (GY), and L875A-N879A-G1001A-Y1003A (LNGY) mutants on GST-H2A C-terminal tail (GST-H2Ac) was compared to that of wild type Bub1^kinase^. Lane 2 is a positive control for monitoring (auto) phosphorylation with the Pro-Q® Diamond Phosphoprotein Gel Stain. Note that in lanes 3 to 10 auto-phosphorylation of the Bub1^kinase^ domain was not observed because the kinase was added is amounts that make it undetectable in this assay (~40 ng kinase against 2 μg substrate were used). (**C**) Kinetic analysis of the indicated Bub1^kinase^ variants using the ADP-Glo^TM^ Kinase Assay, with the reaction velocity plotted as a function of substrate concentration.
(EPS)

S6 Fig Fitted sedimentation data of AUC experiment. The radial signals of the sedimentation velocity absorption profiles of the Bub1:Bub3 (**A**) and BubR1:Bub3 (**B**) complexes and the corresponding residuals of the fits showing the deviation of the c(S) model from the observed signals.
(TIF)
