# Supplementary figures and images for: Role of Intrinsic and Extrinsic Factors in the Regulation of the Mitotic Checkpoint Kinase Bub1
Source: PLoS One. 2015 Dec 10;10(12):e0144673. doi: 10.1371/journal.pone.0144673 (PMC4675524; doi:10.1371/journal.pone.0144673)

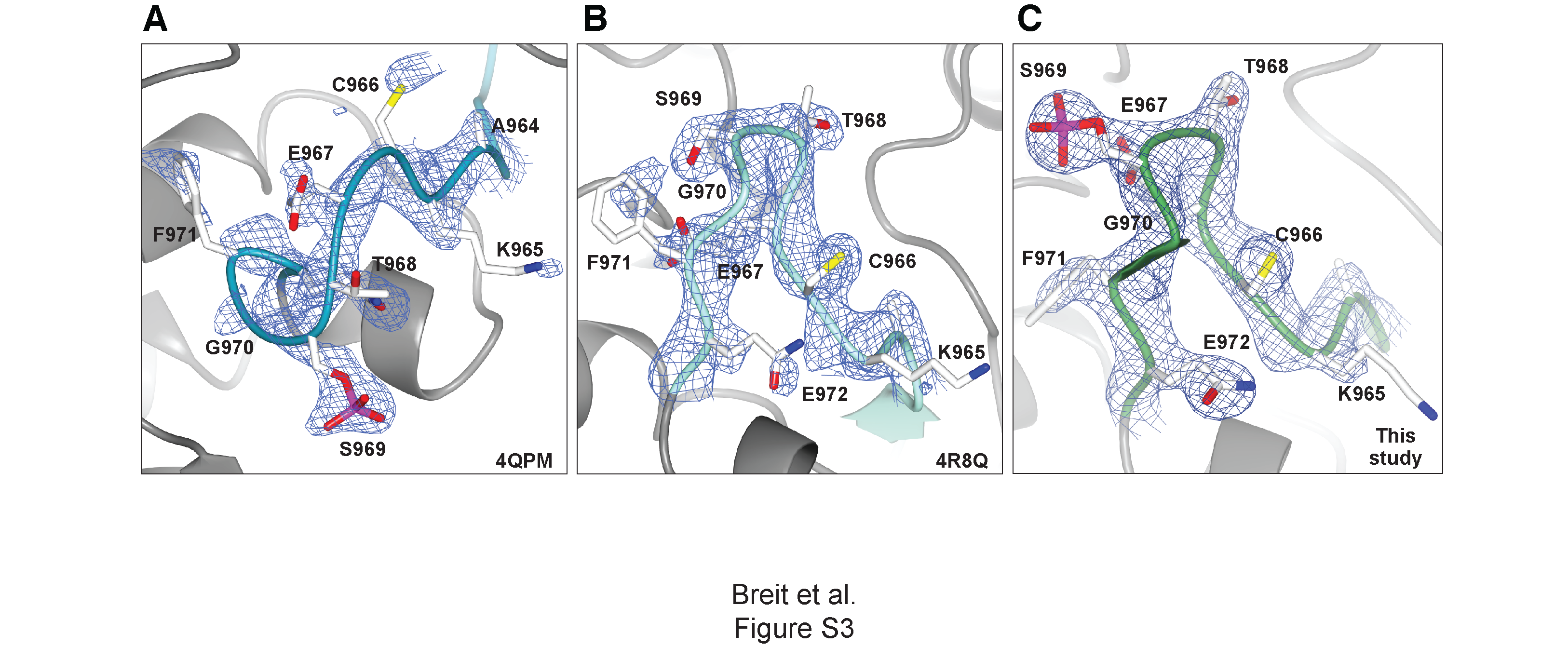

Supplement: S3 Fig — Detailed view of the P+1 loop of (A) phosphorylated Bub1740-1085 (PDB ID 4QPM), (B) unphosphorylated Bub1724-1085 (PDB ID 4R8Q), and (C) Bub1kinase (5DMZ, this study). Side chains are shown as sticks. The electron density around the P+1 loop for 4QPM and 4R8Q has been rebuilt and refined from the structure factors and is represented by the 2Fo-Fc map, contoured at 1.5σ as a blue mesh for all structures. (TIF) [file pone.0144673.s003.tif]

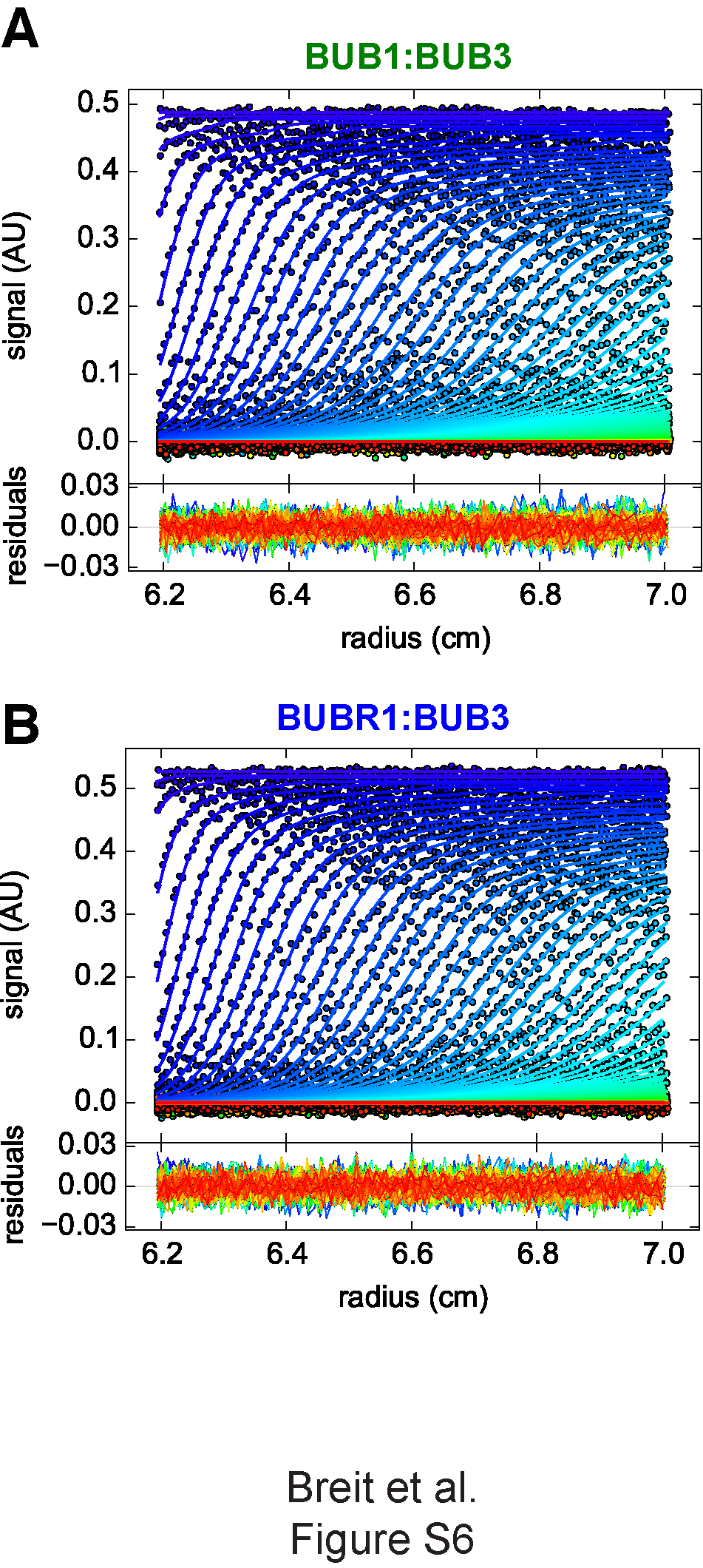

Supplement: S6 Fig — The radial signals of the sedimentation velocity absorption profiles of the Bub1:Bub3 (A) and BubR1:Bub3 (B) complexes and the corresponding residuals of the fits showing the deviation of the c(S) model from the observed signals. (TIF) [file pone.0144673.s006.tif]
